# Pro-Environmental Behavior in an Aging World: Evidence from 31 Countries

**DOI:** 10.3390/ijerph18041748

**Published:** 2021-02-11

**Authors:** Yan Wang, Feng Hao, Yunxia Liu

**Affiliations:** 1 Department of Sociology, Zhou Enlai School of Government, Computational Social Science Lab, Nankai University, Tianjin 300353, China; wang-yan@nankai.edu.cn; 2 Department of Sociology, University of South Florida, Tampa, FL 33620, USA; fenghao@sar.usf.edu; 3 Zhou Enlai School of Government, Nankai University, Tianjin 300353, China

**Keywords:** environmental behavior, age, population aging, multilevel research

## Abstract

Population change and environmental degradation have become two of the most pressing issues for sustainable development in the contemporary world, while the effect of population aging on pro-environmental behavior remains controversial. In this paper, we examine the effects of individual and population aging on pro-environmental behavior through multilevel analyses of cross-national data from 31 countries. Hierarchical linear models with random intercepts are employed to analyze the data. The findings reveal a positive relationship between aging and pro-environmental behavior. At the individual level, older people are more likely to participate in environmental behavior (*b* = 0.052, *p* < 0.001), and at the national level, living in a country with a greater share of older persons encourages individuals to behave sustainably (*b* = 0.023, *p* < 0.01). We also found that the elderly are more environmentally active in an aging society. The findings imply that the longevity of human beings may offer opportunities for the improvement of the natural environment.

## 1. Introduction

The proportion of people over the age of 65 worldwide has increased from 4.97 percent in 1960 to 9.1 percent in 2019 [1]. Demographic forecasts predict that by 2050 the number will double and there will be one older person in every six people [1]. On the one hand, population aging reflects the progress of public health as well as economic and social development. On the other hand, population has been identified as one of the key driving forces of environmental impacts and greater longevity exerts additional pressure on the planet [2].

Most current studies have recognized the social and economic impacts of population aging, whereas its environmental impacts are largely neglected. In particular, little research addresses how population aging affects individual environmental behavior and among the limited studies, theorization and empirical evidence have been surprisingly mixed [3,4]. Some research suggests that people in old age are more likely to engage in pro-environmental behaviors. The theory of generativity posits that aging involves a reexamination of life roles and a shift towards other-centered orientation as it is associated with increased wisdom and emphasis over the feeling of self-importance and being needed, thus older adults are eager to contribute to society and impart a lasting legacy for themselves and future generations [5,6,7,8]. The elderly may participate in environmental protection as one route of successful resolution of the generativity crisis of getting old [8,9]. In a similar vein, the positive psychology of aging proposes that despite the stereotypical image of decline and losses, there are gains and areas of growth during old adulthood, such as enhanced appreciation of the fragility and beauty of life, which enables them to be better citizens and conservationists [10]. Some argue that the elderly participate in environmental behaviors due to practical concerns as they are more vulnerable to environmental degradation and the potential and ongoing environmental problems are threatening their health [11,12]. In order to protect themselves from environmental risks and to enjoy environmental amenities, they tend to value environmental protection and actively participate in environmentally friendly behavior [13,14]. Depending on the level of social development and personal income level, older people who have experienced times of scarcity or live on a low income tend to set a lower standard in terms of comfort and convenience and be more frugal and careful to avoid wastefulness and conserve resources [15,16].

In contrast to the theory of generativity, the socioemotional selectivity theory proposes that as people age, perceived limitations on time lead to motivational change and shift people’s pursuit of expansive goals that focus on obtaining knowledge or making new social contacts towards emotionally meaningful goals that prioritize current feeling-states over concerns for the future [17,18]. The improvement of environmental problems is reckoned as an analytical, long-term goal that may not necessarily affect current well-being. As a result, older adults are expected to be less interested in acquiring environmental knowledge and participate in fewer sustainable behaviors [19]. A related argument is that compared to the younger generation, older people are less eager to embrace post-materialist values, adopt new sustainable ideas, and obtain environmental knowledge and information [20,21], which may also discourage pro-environmental behaviors. Environmentalism is fundamentally incompatible with the growth logic embedded in the capitalist economic system and is often seen as a threat or a potentially disruptive power to the existing social order [22,23]. Older individuals are more tolerant of the current social system and less willing to deal with the risk and uncertainty associated with innovative environmental ideology and social movements and therefore they tend to endorse a more conservative environmental attitude [22].

There is much less research exploring the effect of population aging at the societal level on individual pro-environmental behavior, and the empirical evidence is tenuous and often equivocal. An aging population indicates that there is an increasing proportion of people who are dependent on economic, medical, social, and environmental resources, which may reduce productivity as a whole and become obstacles towards global sustainability [24]. The rising dependency ratio may lead to less output and curb economic growth and place great pressure on the government budget [25,26,27]. It is found that population aging is positively associated with government health expenditure and therefore the increasing demand for elderly health care in aging societies is likely to cause additional budgetary pressure on governmental environment expenditures [28]. The consequent budgetary pressure may limit stakeholders’ willingness and capability of environmental governance and sustainable development, which is essential in laying out the institutional context that facilitates individual pro-environmental behavior by providing green products and infrastructure, encouraging sustainable culture and norms, and dealing with environmental externalities [29,30].

However, there are also studies questioning whether population aging leads to an overall reduction in pro-environmental behavior. Tonn et al. [14] identified two mechanisms through which population aging promotes environmental policies and encourages individuals to participate in environmental activities. The first mechanism is widespread support for environmental protection across age groups. Environmental protection has become a consensus among the general public. World polity theorists concur that under the influence of the world environmental regime, the spread of environmental discourse and institutional structure generate diffuse influence on collective and individual actors across all levels of the social system, including both young and old generations [31,32]. The second mechanism is that greater longevity encourages people to reduce pollution and preserve the environment for their own well-being. Using an overlapping generations model, Mariani et al. [33] revealed that the extent to which individuals invest in environmental activities is contingent on how long they expect to live. Although currently there is a divergence in government expenditure preference between young and old generations where younger people are in favor of environmental maintenance and healthcare expenditure is supported by the latter, the increased lifespan and the enlarged older population, in the long run, will induce fundamental changes in environmental attitude at the societal level and shift individual and organizational resources towards environmental protection [28,34]. In fact, a study based on a national sample from the US suggests that after views toward the government and climate change are accounted for, older respondents are more likely to vote for pro-environmental policies [35].

This study is an attempt to address the controversy in the emerging literature on the relationship between the dramatic growth in older people and pro-environmental behavior. Based on cross-national data from 31 countries, we assessed the impact of individual and population aging on individual behavior. More specifically, we first investigated whether older people are more likely to participate in pro-environmental behaviors. Secondly, as population aging may influence environmental discourse and policy preferences in a society, we also examined whether population aging at the national level motivates individuals to participate in sustainable behaviors.

## 2. Data and Methods

### 2.1. Design

The current study is a multi-level analysis based on cross-national data from 31 countries. More specifically, we estimated and reported hierarchical linear models with random intercepts. The research design is appropriate because of the nesting data structure.

### 2.2. Data

The individual-level data are obtained from the most recent wave (2010) of the environment module launched by the International Social Survey Programme (ISSP2010) [36]. It consists of nationally representative samples from 32 societies for the year 2010. The ISSP2010 allowed us to examine environmental attitudes and behaviors across different age groups in a variety of social settings. National-level data are from the World Development Indicators (WDI) [37] and Environmental Performance Index (EPI) [38]. All country-level variables are for the year 2010 to be consistent with individual-level variables. We combined these two datasets for the analyses. The original dataset contained 45,199 individuals from 32 societies. Taiwan (*n* = 2209) was not included due to missing data on country-level variables. After excluding observations with missing variables, the final analytical sample consisted of 40,542 individuals in 31 countries (Table 1).

### 2.3. Variables

The ISSP2010 asked respondents about the frequency with which they participate in the following activities for environmental reasons: (1) sort glass or tins or plastic or newspapers and so on for recycling; (2) buy fruits and vegetables grown without pesticides or chemicals; (3) reduce the energy or fuel used at home; (4) choose to save or reuse water; (5) avoid buying certain products; and (6) cut back on driving a car. Answers were given along a 4-point scale, ranging from 1 (always) to 4 (never). As long as around one-quarter of the respondents did not own or could not drive a car, the last item was not included in the analysis. The dependent variable, pro-environmental behaviors, is a composite index of the first five items. To create the dependent variable, we reverse coded the variables so that higher values indicated higher levels of pro-environmental behaviors and then took their average (Cronbach alpha = 0.74).

The key independent variable, age, is a continuous variable measured by respondents’ self-reported age. Additionally, people across age groups may exhibit different levels of pro-environmental behaviors. For example, younger people, middle-aged people, and older people may have different behavioral patterns, and within each generation, there could also be nuanced variations. By treating age as one continuous variable, we can only reveal a linear relationship between age and pro-environmental behavior and may run the risk of not capturing more complicated effects. Demographers conventionally consider 65 as the start of old age. More specifically, the elderly are further classified into three groups, the youngest-old (65–74), the middle-old (75–84), and the oldest-old (85 and above), and each group has different physical and psychological conditions [39]. Therefore, we also included a set of binary variables (15–24, 25–34, 35–44, 45–54, 55–64, 65–74, 75–84, and 85 and above) to examine the presence of systematic patterns, potential curvilinear relationships, as well as variations within the elderly. Following the standard approach, population aging at the national level is measured by the percentage of population aged 65 and above in the total population in the country [1].

Individual-level control variables include environmental concern, environmental efficacy, environmental knowledge, gender, and educational level. Previous studies suggested that environmental attitudes are important predictors of environmental behaviors [40,41]. Environmental concern is one of the most widely identified factors that influence pro-environmental behaviors [42,43]. We use the following survey question from the ISSP2010 to evaluate environmental concern: “Generally speaking, how concerned are you about environmental issues?” Participants responded on a 5-point scale (from 1 = not at all concerned to 5 = very concerned).

Environmental efficacy was first proposed in Ajzen’s [44] theory of planned behavior as the evaluation of resources and opportunities available to the person that dictates the actual impact of intended behavior. Its effect on pro-environmental behaviors is well-supported by empirical evidence [45,46]. In the current study, environmental efficacy is derived from the respondents’ agreement on six statements in the ISSP2010: (1) it is too difficult for someone like me to do much about the environment; (2) I do what is right for the environment, even when it costs more money or takes more time; (3) there are more important things to do in life than protect the environment; (4) there is no point in doing what I can for the environment unless others do the same; (5) many of the claims about environmental threats are exaggerated; and (6) I find it hard to know whether the way I live is helpful or harmful to the environment. The values range from 1 (disagree strongly) to 5 (agree strongly). We reverse coded the second item and then calculated the average value (Cronbach alpha = 0.67). Considering that this measure of environmental concern might be influenced by the respondents’ actual pro-environmental behavior, it may reduce the informational value of this variable and lead to confounding errors. Therefore, we examined the correlation between these two variables and the correlation coefficient is 0.31, suggesting that the correlation is not very high and that the respondents’ answers to these survey questions may not necessarily strongly influence each other. Additionally, this measurement is widely used in the literature when predicting pro-environmental behavior and has been proven to be a valid construct [47,48].

The possession of environmental knowledge plays an important role in motivating individuals to act environmentally friendly [49]. ISSP2010 asked respondents to evaluate the extent to which they feel they know about the (1) causes of and (2) solutions to a series of environmental problems, including air pollution, chemicals and pesticides, water shortage, water pollution, nuclear waste, domestic waste disposal, climate change, genetically modified foods, and use of natural resources. The answers are measured using a 5-point scale varying from 1 (know nothing at all) to 5 (know a great deal). The variable was obtained from these statements by taking the average (Cronbach alpha = 0.81).

Gender was measured using a dummy variable (female = 1). Educational level was measured using four dichotomous variables: less-than-secondary qualification, intermediate secondary education completed, higher secondary education completed, and university degree (incomplete or completed).

At the national level, we controlled for economic development, total population, population density, and air pollution. Individual pro-environmental behavior is constantly found to be influenced by the level of economic development through post-materialist values, pollution transfer, environmental politics, and other mechanisms [50,51]. Additionally, changes in population age structure first occur in affluent societies and are closely associated with the level of economic development [52]. As a result, economic development is accounted for in the model, measured by gross domestic product (GDP) per capita in constant 2005 US dollars. Following previous studies, e.g., [53], this study used residual GDP per capita on population aging to reduce multicollinearity. Human ecologists argue that the size and density of population significantly influence ecological capacity and lead to environmental impacts [54,55], and therefore we assessed their effects in the analyses.

Finally, the degradation hypothesis suggests that the presence of objective environmental problems results in direct experiences of citizens with environmental deterioration and increases the perceived threat [56,57]. Since environmental issues pose greater health risks for senior citizens, we included the level of PM_2.5_ as an indicator of environmental quality in the country. Compared to other forms of environmental issues, air pollution, especially the one caused by PM_2.5_ particles, is more visible to the general public and also gives rise to cardiovascular and respiratory diseases [58,59]. Except for the level of PM_2.5_ which is obtained from the EPI data, all other country-level variables were obtained from the WDI data. All national-level variables were logged to address their positively skewed distribution.

### 2.4. Analytical Methods

We employed multilevel modeling techniques to assess the extent to which individual-level pro-environmental behavior is associated with aging at the individual and national levels due to the nesting data structure [60]. Since the outcome variable is a continuous variable and there are no theoretical reasons to assume that each country has a separate regression model with its own intercept and slope, we estimated hierarchical linear models with random intercepts. To better identify cross-level interactions, the age variable at the individual level was group mean centered. All national-level predictors and the non-dichotomous control variables at the individual level were grand mean centered [61].

## 3. Results

Table 2 presents descriptive statistics for the individual-level and country-level variables. The mean value of pro-environmental behavior is 2.37 out of a 4-point scale, suggesting that people on average have a moderate level of pro-environmental behavior. The average age in the sample is 47 years old and 19% are above 65 years old. At the national level, the mean value of aging population is 14%.

We estimated the null model without predictors (results available upon request). The national-level variance is 0.079 and the interclass correlation is 0.180 (*p* < 0.001), indicating that 18% of the variance in pro-environmental behavior is between countries. As a result, it is necessary to examine national-level predictors for a better understanding of individual pro-environmental behavior.

The findings for the multilevel linear models are presented in Table 3. Model 1 consists of all individual- and national-level control variables and serves as the baseline model. Consistent with previous studies based on cross-national data [62,63], all three measures of environmental attitudes are positively associated with pro-environmental behavior, and females are more likely to act environmentally than males. It was interesting to find that people with the lowest level of education, i.e., less than secondary qualification, perform more actively in pro-environmental behavior than those with higher educational levels. However, later we found out that this is not actually the case because the effects of age and education mix in the current model. In other words, older individuals on average have lower education than younger generations and they participate in more pro-environmental behaviors. Among the national-level predictors, only population density significantly impacts the outcome variable, and higher population density is positively associated with more pro-environmental behavior at the individual level. Considering that there are only 31 cases at the country level, we flagged statistical significance up to the 0.10 level. Results suggest that GDP per capita has a marginally significant effect on pro-environmental behavior, and population size and PM_2.5_ level do not have significant influences, net of other variables.

Model 2 adds the age variable to assess the impact of age after accounting for the control variables. On average, age is positively associated with pro-environmental behavior. Older people are more likely to participate in pro-environmental behavior. More specifically, a ten-year increase in age is associated with a 0.52-point increase in pro-environmental behavior on a 4-point scale (*p* < 0.001). Additionally, after age is included in the model, the effects of other variables are basically the same, while educational level becomes positively related to pro-environmental behavior, which is consistent with past research.

**Figure 1:**
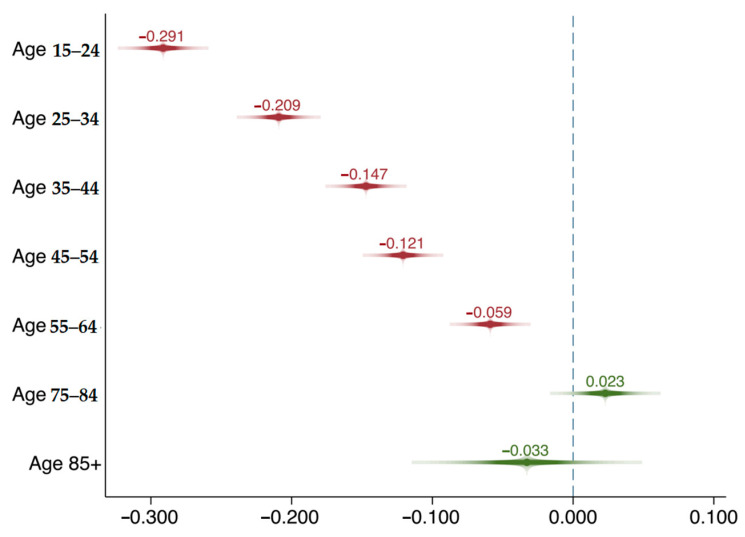
The predicted effect of age on pro-environmental behavior, ISSP2010. This figure is based on Model 3, Table 3. The reference group is people aged 65 to 74. The numbers above the lines represent the predicted effect size. The red lines indicate significant results (*p* < 0.05) and the green lines indicate non-significant results.

Model 3 replaces the continuous measure of age with a series of age categories to examine if there are curvilinear relationships between age and pro-environmental behavior. The other variables remain the same. The youngest-old individuals aged 65 to 74 years (i.e., the reference group) are the most active in participating in pro-environmental behaviors, and there are no significant differences among the youngest-old (ages 65 to 74), middle-old (ages 75 to 84), and oldest-old (ages 85 and above) individuals. The positive association between age and the outcome variable in Model 2 is largely due to the increase in pro-environmental behavior as people become old (Figure 1).

Table 4 shows the estimated effect of population aging at the national level on individual pro-environmental behavior. Model 4 includes country-level population aging in addition to all variables in Model 3. Compared to Model 2, after accounting for population aging, the model explains an additional 22% ((0.044 − 0.034)/0.044) of the cross-national variance in pro-environmental behavior. The results suggest that population aging significantly promotes individual pro-environmental behavior. As mentioned earlier, this is likely due to the spread of environmental discourse and organizational settings under the world environmental regime and the emphasis on individual wellbeing associated with a sustainable environment.

Model 5 includes a cross-level interaction term to examine the extent to which aging at the micro-and macro-levels together shapes pro-environmental behavior. The interaction term is positive and statistically significant (*p* < 0.001), suggesting that the positive relationship between aging at the individual level and pro-environmental behavior is strengthened in an aging society.

Two sensitivity tests were conducted to evaluate the robustness of the findings reported here. Firstly, we included country dummies in the models. Secondly, considering that there might be variations across different kinds of pro-environmental behaviors, we run models predicting each pro-environmental behavior that constructs the dependent variable. For both sensitivity tests, the results are basically the same as the ones we reported (results available upon request).

## 4. Discussion

Our findings confirm that aging at both individual and national levels promotes individual pro-environmental behavior and therefore help to settle the ongoing dispute. More specifically, we found a positive relationship between age and pro-environmental behaviors at the individual level. This is consistent with the positive arguments such as the theory of generativity and positive psychology of aging [6,7,8,10]. As people grow old, they may increasingly seek self-transcendence meaning of life and pursue pro-social goals, and thus the practice of environmentally friendly behaviors may become one route for older adults to impart such wisdom and stay active. The enhanced perceived impact of environmental risks on human health may also encourage older people to actively engage in environmental issues to prevent and decrease environmental threats. Furthermore, people of old age may be more likely to participate in pro-environmental behaviors such as resource conservation and rational shopping out of habits or financial constraints. All of these could explain the positive relationship between age and environmental behaviors. It should also be noted that research reports that younger generations have higher levels of pro-environmental attitudes. This could be explained by the so-called environmental “attitude–behavior gap” [40]. Younger people may claim that they care more for the environment compared to their older counterparts, but sacrifice in terms of convenience and costs often becomes a barrier that prevents the transition from environmentally friendly attitudes to actual behaviors [64].

The results also suggest that population aging at the national level promotes individual environmental behaviors. Since older people on average act more sustainably, their growing number may collectively contribute to a more environmentally friendly public discourse and encourage individuals to participate more in environment-conscious activities. From the perspective of public policy, the growing discourse on the environment is likely to stimulate environmental governance among different stakeholders and promote government, enterprises, and other relevant parties to invest in environmental issues. As a result, the increase in governmental health expenditures due to population aging is not necessarily in conflict with environmental expenditures. In addition, we found that aging at the individual and national level produces a multiplicative impact on environmental behavior. In other words, older adults have a higher probability of practicing environmental actions when they live in an aging society.

Regarding the effects of control variables, consistently with previous studies, females are more likely to participate in pro-environmental behaviors due to their higher levels of environmental concern and post-materialist values [65,66]. After age is included in the models, as in previous research [62,67], education appears to have a marginally positive relationship with pro-environmental behavior. Economic development is found to facilitate individual environmental participation through the spread of post-materialist values and increasing environmental concern. Our findings lend partial support to this argument. Previous studies noted the importance of population in predicting pro-environmental behavior; our research also found a positive relationship between population density and individual behavior. It is interesting to see that contrary to the degradation hypothesis, the level of PM_2.5_ does not influence individual environmental engagement. In fact, evidence for the effect of air pollution on individual environmental practice has been mixed in the literature [68,69]. Since it is not the focus of the current research, we did not delve into this issue. Future research should explore the mechanisms through which objective environmental quality impacts individual behavior.

Our analyses advance the emerging body of research on the intersection between population aging and environmental sustainability by examining the impacts of population aging at the individual and national levels on pro-environmental behaviors. But the current research still has several limitations. Firstly, this study does not distinguish the age effect from the cohort effect. Age is a physical, psychological, and sociological construct in that it not only indicates the life course stage, but also shows cohort cultural differences [3,22]. Earlier studies use education and ideology as indicators of cohort effect, e.g., [22]. Although educational level has been controlled for in the models, it does not necessarily rule out all the historical and social experiences shared among cohorts. Secondly, the self-reported environmental behaviors used in this study do not assess the respondents’ actual environmental impact. Current research has developed two approaches to evaluate environmental behavior: intent-oriented and impact-oriented approaches [70]. The intent-oriented approach cannot precisely evaluate the environmental externalities of particular behaviors, as the intended and actual impact can differ considerably [71]. Thirdly, we focused on pro-environmental behavior in the private sphere and did not investigate public environmental behavior. Public environmental behavior includes actions such as volunteering and donating for environmental issues. Previous research suggests that older people’s participation in environmental volunteering can effectively lessen volunteer shortage and at the same time offer opportunities for social integration in later life [9]. Future analyses would benefit from the examination of the effect of aging on public environmental behavior.

Despite the limitations, this research contributes to the current knowledge in three important ways. Firstly, it provides evidence for the ongoing debate over how population aging affects individual environmental behavior. The cross-national multilevel dataset enabled us to assess the extent to which aging at the individual and national levels influences pro-environmental behavior in the international context. Secondly, our findings help to enrich the understanding of sustainability by highlighting a sustainable environment restored and protected by older people, which connects older generations with future generations and connects human beings with nature. The elderly can become a valuable resource rather than a threat to sustainable development. Thirdly, this current research design also broadens the scope of environmental gerontology. Environmental gerontology typically focuses on how natural and built environment is related to the wellbeing and longevity of older people and previous studies suggested that a sustainable environment is necessary for healthy aging. We argue that the benefits are in fact bidirectional and that older individuals play an important role in environmental conservation as well.

## 5. Conclusions

Population change and environmental degradation have become two of the most pressing issues in the contemporary society, but the effect of population aging on the environment remains unclear. The current study attempts to fill this gap in the literature by systematically examining the impact of aging at the individual as well as national levels on pro-environmental behavior. At the individual level, the results suggest that older individuals are more likely to participate in pro-environmental behaviors and there are no significant differences within the senior group. At the national level, population aging also facilitates individual pro-environmental behavior.

Population aging has become an irreversible and overwhelming trend in the world. The findings of this paper shed light on how population aging can be compatible with environmental sustainability. Traditionally, older people are often perceived to consume a disproportionate amount of environmental resources and space. However, this study in fact reveals that they are also active actors in resource conservation and environmental protection and their increasing number makes them an indispensable part of solutions to environmental problems. In this sense, the longevity of human beings offers opportunities for the maintenance of the natural environment in the long run. The benefits are bidirectional at the individual, community, and societal levels. On the one hand, older persons’ environmental participation improves environmental quality and reduces potential threats. On the other hand, exposure to a sustainable and pleasant environment promotes the physical and psychological health of the older population as well as the wellbeing of society as a whole.

## Figures and Tables

**Table 1:** Countries included in the study.

Countries Included in the Study			
Argentina	Denmark	Lithuania	South Africa
Austria	Finland	Mexico	Spain
Belgium	France	New Zealand	Sweden
Bulgaria	Germany	Norway	Switzerland
Canada	Israel	Philippines	Turkey
Chile	Japan	Russian Federation	United Kingdom
Croatia	Korea, Rep.	Slovak Republic	United States
Czech Republic	Latvia	Slovenia	

**Table 2:** Descriptive statistics.

**Continuous Variables**	**Mean**	**SD**	**Range**
Dependent variable			
Pro-environmental behavior	2.37	0.72	1–4
Individual-level predictors			
Age	47.13	17.43	15–99
Environmental concern	3.61	1.12	1–5
Environmental efficacy	3.14	0.70	1–5
Environmental knowledge	2.91	0.97	1–5
National-level predictors			
Population aging (% population ≥ 65)	14.34	4.57	4.14–22.50
GDP per capita	25,441.06	17,565.01	1403.38–66,117.01
Population size	45,400,000	63,700,000	2,048,583–309,000,000
Population density (people/km^2^)	127.60	130.66	3.75–508.86
PM_2.5_ (mg/m^3^)	6.46	2.97	0.35–13.83
**Categorical Variables**		**Percentage**	***n***
Individual-level predictors			
Age 15–24		11%	4611
Age 25–34		17%	6691
Age 35–44		18%	7474
Age 45–54		18%	7297
Age 55–64		17%	6821
Age 65–74		12%	5037
Age 75–84		6%	2230
Age 85 and above		1%	381
Gender (female = 1)		54%	21,928
Less than secondary qualification		20%	8011
Intermediate secondary education completed		22%	8776
Higher secondary education completed		27%	10,944
University degree (incomplete and completed)		32%	12,811

Notes: *n* = 40,542 (individuals); *N* = 31 (countries).

**Table 3:** Multilevel linear models predicting pro-environmental behavior, ISSP2010.

	Model 1	Model 2	Model 3
Age		0.052 ***	
		(0.002)	
Age groups (Ref. age 65–74)			
Age 15–24			−0.291 ***
			(0.013)
Age 25–34			−0.209 ***
			(0.012)
Age 35–44			−0.147 ***
			(0.011)
Age 45–54			−0.121 ***
			(0.011)
Age 55–64			−0.059 ***
			(0.011)
Age 75–84			0.023
			(0.015)
Age 85 and above			−0.033
			(0.032)
Individual-level control variables			
Environmental concern	0.113 ***	0.109 ***	0.109 ***
	(0.003)	(0.003)	(0.003)
Environmental efficacy	0.194 ***	0.195 ***	0.194 ***
	(0.005)	(0.005)	(0.005)
Environmental knowledge	0.085 ***	0.088 ***	0.088 ***
	(0.004)	(0.003)	(0.003)
Gender (female = 1)	0.093 ***	0.095 ***	0.095 ***
	(0.006)	(0.006)	(0.006)
Intermediate secondary education completed (ref.: less than secondary qualification)	−0.042 ***	0.015	0.011
	(0.010)	(0.010)	(0.010)
Higher secondary education completed	−0.057 ***	0.019 *	0.017
	(0.009)	(0.010)	(0.010)
University degree (incomplete and completed)	−0.050 ***	0.020 *	0.015
	(0.010)	(0.010)	(0.010)
National-level control variables			
GDP per capita (logged and residual)	0.087	0.085	0.083
	(0.052)	(0.052)	(0.050)
Total population (logged)	0.033	0.036	0.037
	(0.030)	(0.030)	(0.029)
Population density (logged)	0.106 **	0.107 **	0.107 **
	(0.038)	(0.038)	(0.036)
PM_2.5_ (mg/m^3^)	−0.022	−0.022	−0.022
	(0.017)	(0.017)	(0.016)
Intercept	2.376 ***	2.319 ***	2.447 ***
	(0.039)	(0.039)	(0.038)
National-level variance	0.043 ***	0.044 ***	0.041 ***
Log-likelihood	−37,020.506	−36,622.008	−36,608.413

Notes: *n* = 40,542, *N* = 31; standard errors in parentheses; *** *p* < 0.001, ** *p* < 0.01, * *p* < 0.05

**Table 4:** Multilevel linear models predicting the effect of population aging on pro-environmental behavior, ISSP2010.

	Model 4	Model 5
Age	0.052 ***	0.051 ***
	(0.002)	(0.002)
Population aging	0.023 **	0.023 **
	(0.008)	(0.008)
Age × population aging		0.003 ***
		(0.000)
Individual-level control variables		
Environmental concern	0.109 ***	0.109 ***
	(0.003)	(0.003)
Environmental efficacy	0.195 ***	0.196 ***
	(0.005)	(0.005)
Environmental knowledge	0.089 ***	0.088 ***
	(0.003)	(0.003)
Gender (female = 1)	0.095 ***	0.095 ***
	(0.006)	(0.006)
Intermediate secondary education completed (ref.: less than secondary qualification)	0.014	0.009
	(0.010)	(0.010)
Higher secondary education completed	0.019	0.013
	(0.010)	(0.010)
University degree (incomplete and completed)	0.020 *	0.016
	(0.010)	(0.010)
National-level control variables		
GDP per capita (logged and residual)	0.082	0.082
	(0.046)	(0.046)
Total population (logged)	0.058 *	0.058 *
	(0.027)	(0.027)
Population density (logged)	0.086 *	0.086 *
	(0.034)	(0.034)
PM_2.5_ (mg/m^3^)	−0.015	−0.015
	(0.015)	(0.015)
Intercept	2.313 ***	2.317 ***
	(0.035)	(0.035)
National-level variance	0.034 ***	0.034 ***
Log-likelihood	−36,618.386	−36,582.744

Notes: *n* = 40,542, *N* = 31; standard errors in parentheses; *** *p* < 0.001, ** *p* < 0.01, * *p* < 0.05.

## Data Availability

The data presented in this study are available on request from the corresponding author.
